# Evaluation of AI-Assisted Stethoscope for Cardiac Time Intervals in Pediatric Patients: A Viable Echocardiographic Alternative

**DOI:** 10.1016/j.jacadv.2024.101078

**Published:** 2024-07-31

**Authors:** Eli S. Fredman, Nathan Miller, Lauren M. Littell, Ava Claire Lariego, William B. Orr, Tracy M. Conner, Anthony Pompa, Lisa Roelle, Ram K. Rohatgi, Jennifer A. Silva

**Affiliations:** a Washington University School of Medicine, St Louis, Missouri, USA; b St Louis Children’s Hospital, St Louis, Missouri, USA; c Arizona State University, Tempe, Arizona, USA; d Washington University McKelvey School of Engineering, St Louis, Missouri, USA

Cardiac auscultation, vital in resource-limited settings, has been overshadowed by diagnostic advancements in echocardiography. The advent of stethoscope technology and artificial intelligence (AI) has revitalized its importance, particularly for detecting subtle findings such as cardiac time intervals (CTIs). AI-enhanced digital stethoscopes are promising for early heart failure detection, highlighting the potential of CTIs in patient monitoring and disease management.^1^^,^^2^

Pediatric heart failure patients exhibit longer systolic times (St) and increased systolic-to-diastolic ratios (St/Dt) compared to healthy children.^3^ In congenital heart disease, pulmonary hypertension, or cardiomyopathies, changes in St/Dt are associated with increased morbidity. Despite CTI’s utility, echocardiographic assessment is limited by inconvenience and resource utilization.^4^

AI-assisted stethoscope data offer a cost-effective, accessible method for measuring CTIs, suitable for integration into clinical practice, telemedicine, and long-term patient monitoring.

This study assesses the reliability of AI-assisted stethoscope in quantifying CTIs in children without structural heart disease, considering variations in heart rate and age. Subjects with structurally normal hearts undergoing electrophysiology study and ablation were consented/assented for the study, per Institutional Review Board protocol.

During the electrophysiology study post-ablation waiting phase, CTIs were measured using a Stethee Pro stethoscope (M3DICINE) and Epiq ultrasound system (PHILIPS Medical). Measurements were taken consecutively under 3 conditions: baseline heart rate (BHR), atrial pacing at BHR +10 beats/min, and BHR + 25 beats/min. Over a 20-second period, the stethoscope recorded each cardiac cycle, with its AI software automatically averaging, segmenting, and classifying St, Dt, and total cardiac cycle (TCC) duration using its proprietary deep neural network model. Echocardiography captured TDI waveforms and were analyzed according to previously described methods by 2 cardiologists to ensure inter-user consistency.^4^^,^^5^

Intraclass correlation coefficient (ICC) assessed agreement between stethoscope and echocardiogram, and between the 2 readers, and was classified as poor (<0.50), moderate (0.50-0.75), good (0.75-0.90), or excellent (>0.90). Mixed effects regression models with random subject effect and variance component covariance structure analyzed CTI and HR relationships. HR was included in the unadjusted model as the fixed effect. Adjusted analyses were performed with adjustment for additional covariates in the mixed effects model, including age, sex, and body surface area. Mixed effects model assumptions were checked by residual plots and the assumption held for all performed mixed effects model. Analysis was performed using SAS, version 9.4 (SAS Institute Inc).

Analysis included 22 subjects (female 45%, age 12.8 ± 5 years, resting HR 94 ± 15 beats/min), showing moderate/good reliability between both stethoscope and echocardiogram (ICC St: 0.65; Dt: 0.76; TCC: 0.76; St/TCC: 0.57; St/Dt 0.57) and interobserver reliability (ICC St: 0.80; Dt: 0.78; TCC: 0.82; St/TCC: 0.67; St/Dt: 0.60).

**Figure 1:**
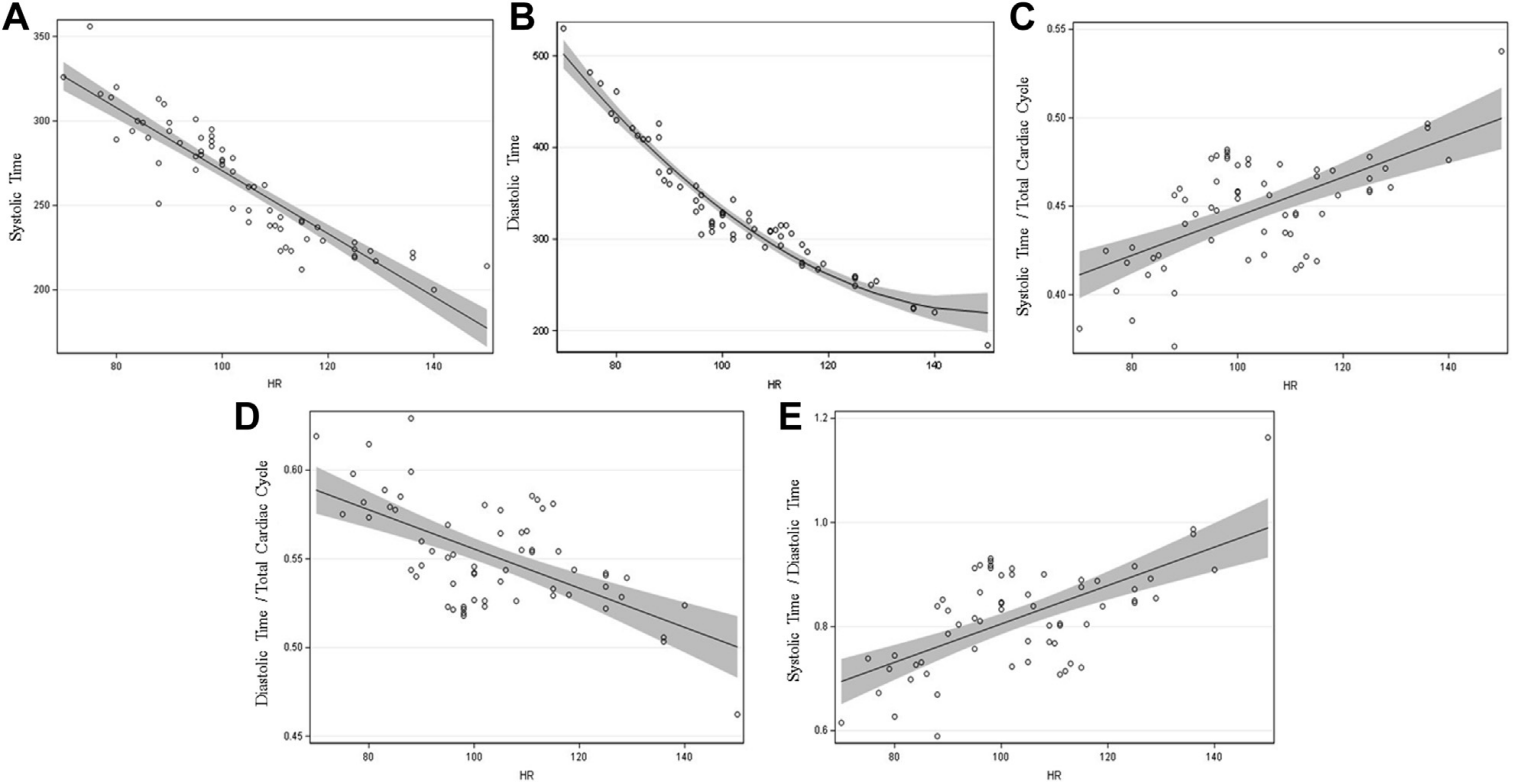
**Cardiac Time Intervals vs HR** Trend line (continuous line) and upper and lower 95% prediction intervals (shaded region) for St (A); Dt (B); St/TCC (C); Dt/TCC (D); and St/Dt (E). Dt = diastolic time; St = systolic time; TCC = total cardiac cycle.

In the primary unadjusted analysis, model parameters indicate a negative linear relationship between HR and St (−1.87 ± 0.11 ms) and Dt/TCC (0.37% ± 0.06%). There is a significant, positive linear relationship between HR and St/TCC (0.1% ± 0.02%) and St/Dt (0.37% ± 0.06%). There is a significant nonlinear relationship between HR and Dt (0.04 ± 0.006 msec) (Figure 1).

When adjusting for age, sex, and body surface area, the trend of the unadjusted model does not change, although age carries a statistically significant inverse association with St/TCC, Dt/TCC, and St/Dt. Age has a significant, albeit small, relationship with St, indicating a decrease in St with increasing age independent of HR.

This study provides evidence supporting the reliability of AI-assisted stethoscope in CTI measurement, revealing clinically negligible differences compared to echocardiogram measurements, and aligning with previously established norms for changes in CTIs with HR. As HR increases, the linear decrease in St and the curvilinear decrease in Dt underscore the significance of the heart’s capacity to adjust Dt relative to St to influence cardiac output at elevated HRs. Age independently influenced St but not Dt, implying HR might be stronger determinant of Dt dynamics. These insights enhance our comprehension of cardiac physiology, demonstrating the nuanced roles of age and HR in cardiac performance.

The positive linear association between St/Dt and HR and the age-related influences on St/Dt, St/TCC, and Dt/TCC align with previously established pediatric norms. In contrast, prior studies have shown that cardiac dysfunction leads to a higher-than-expected St/Dt, likely owing to the diastolic shortening beyond what would be expected with increasing HR.^3^, ^4^, ^5^

Due to resource constraints, the study was structured as an exploratory analysis with the aim of generating preliminary data and effect size estimates that could inform future studies rather than to generate new normative data. Therefore, while additional exploratory analysis indicated differences in CTIs at various pacing levels, due to the limited study power, the specific effect of pacing could not be quantified.

Our study indicates that AI-assisted stethoscope can accurately quantify CTIs and offers an alternative method compared to echocardiogram, without limitations of interobserver variability and image quality. This provides a framework for future studies in populations with heart disease and may provide insight into longitudinal management of patients.
